# The impact of the learner role in healthcare simulation-based education on learning outcomes: a systematic review

**DOI:** 10.3352/jeehp.2026.23.24

**Published:** 2026-08-06

**Authors:** Abdelkader Amechghal, Aziz Naciri, Kamal Takhdat, Sana Loubbairi, Adil Mellouki, Laila Lahlou, Hicham Nassik

**Affiliations:** 1 Research and Innovation Laboratory in Health Science, Faculty of Medicine and Pharmacy, Ibn Zohr University, Agadir, Morocco; 2 Higher Institute of Nursing Professions and Health Techniques, Laayoune, Morocco; 3 Higher Institute of Nursing Professions and Health Techniques, Marrakech, Morocco; 4 Faculty of Medicine and Pharmacy, Ibn Zohr University, Laayoune, Morocco; Hallym University of Korea, Korea

**Keywords:** Medical education, Educational measurement, Observational learning, Simulation training, Morocco

## Abstract

**Purpose:**

This review examined the impact of learner role—active participant versus observer—on learning outcomes in healthcare simulation-based education using Kirkpatrick’s evaluation model.

**Methods:**

Five databases—PubMed, Scopus, Web of Science, ScienceDirect, and EBSCOhost—were searched for studies published from January 2018 to November 2024. Eligibility criteria, defined using the PICOS framework, targeted studies comparing learning outcomes between active participant and observer roles among health professions students and practitioners. Methodological quality was appraised using the Medical Education Research Study Quality Instrument, and findings were synthesized narratively. This review was registered in PROSPERO under registration number CRD42024611988.

**Results:**

Of 648 records, 15 studies involving 1,799 participants met the inclusion criteria; role allocation was specifically reported for 644 active participants and 709 observers. Most studies were conducted in high-income countries (13/15) and nursing programs (11/15). At Kirkpatrick Level 1, most studies reported no statistically significant between-role differences, although an active participant advantage emerged for emotional arousal. At Level 2, knowledge, skills, and most attitudinal outcomes did not differ significantly between roles; active participant advantages were observed for technical skills at delayed follow-up, affective competencies in palliative care, and perceived learning transfer. No study assessed Levels 3 or 4.

**Conclusion:**

Most studies found no significant differences between observer and active participant roles at Kirkpatrick Levels 1 and 2. Generalizability across all health professions and settings is limited. Pedagogically appropriate use of the observer role supports learning outcomes and may expand access to simulation-based education.

## Graphical abstract

[Fig f2-jeehp-23-24]

## Introduction

### Background

Simulation-based education (SBE) has become an essential pedagogical strategy in healthcare education, enabling learners to acquire and refine clinical knowledge and skills in controlled, safe environments without compromising patient safety. Despite its widespread implementation, the optimal design features of healthcare SBE remain underexplored, including the role assigned to learners during simulation experiences [1]. One key design variable is learner role assignment: the active participant role versus the observer role. Whereas active participants engage directly in the simulation scenario, observers follow the scenario from the same room or remotely via audiovisual transmission; their observation may be supported by structured observation tools or specific instructions, referred to as directed observation [2].

SBE is grounded in theoretical frameworks that guide instructional design and support learning in rare or high-risk clinical situations [3]. Traditionally, SBE has relied on Kolb’s experiential learning theory, which supports active participation as the basis of learning through directed experience followed by reflection [4]. However, Bandura’s social cognitive theory, which underpins observational and vicarious learning, remains underexplored in the simulation literature [5]. This theory posits that learners can develop knowledge and skills by observing others through 4 sequential processes: attentional focus, retention, behavioral reproduction, and motivation [6]. To bridge this theoretical gap, Johnson [7] proposed the observational experiential learning framework, which integrates experiential learning, social learning, and social cognitive theories to support the use of the observer role during SBE. This framework posits that learners can initially acquire knowledge through either active participation or structured observation and subsequently transform this experience into clinical skills through reflection and conceptualization during debriefing.

Healthcare SBE is facing increasing operational demands: student enrollment continues to grow, and curricula are expanding and diversifying [2,8,9]. In parallel, available simulation sites and trained faculty may be insufficient to meet these educational needs [10]. This discrepancy highlights the need to examine alternative teaching strategies, including role assignment during SBE. The observer role may represent a practical and educationally sound approach to extending learning opportunities, particularly in contexts with limited simulation resources. However, the educational impact of the observer role compared with active participation remains unclear.

### Objectives

This systematic review examined the impact of learner role assignment—active participant versus observer—on learning outcomes in healthcare SBE among health professions students and practitioners, classified according to Kirkpatrick’s 4-level evaluation model [11].

## Methods

### Ethics statement

This study did not use materials obtained from human participants; therefore, ethics committee approval and informed consent were not required.

### Study design

This systematic review was conducted according to the Preferred Reporting Items for Systematic Reviews and Meta-Analyses (PRISMA) statement [12]. The review protocol was preregistered in the International Prospective Register of Systematic Reviews (PROSPERO) under the ID CRD42024611988.

### Eligibility criteria

The PICOS framework (Patient, Problem or Population; Intervention; Comparison, Control, or Comparator; Outcome; and Study Design) was used to define the selection criteria and identify pertinent studies (Table 1).

### Information sources

We systematically searched the following electronic databases: PubMed, Scopus, Web of Science, ScienceDirect, and ERIC via EBSCOhost. The search was limited to original, peer-reviewed articles published in English between January 2018 and November 2024. The final search was conducted on November 29, 2024.

### Search strategy

Two authors (A.A. and H.N.) worked together to define the search terms, keywords, and Boolean operators for each database. The complete search strategy is available in Supplemental Digital Content (Supplement 1).

### Selection process

Duplicate records were removed using Rayyan [13]. Two authors (A.A. and H.N.) independently screened all retrieved records by title and abstract, followed by full-text assessment of eligible records against predefined inclusion and exclusion criteria. Disagreements were resolved through discussion with a third author (K.T.).

### Data collection process

One author (A.A.) extracted data from all included studies. Outcome classification according to the 4 levels of the Kirkpatrick evaluation model was performed by consensus among the review authors (A.A., A.N., K.T., S.L., A.M., and H.N.) [11]. Disagreements regarding the interpretation or presentation of statistical results were resolved by an author with statistical expertise (L.L.).

### Data items

Table 2 summarizes the characteristics and findings of the 15 included studies. Extracted data encompassed the following elements: author(s) and year of publication, country, study design, theoretical framework, participants and sample size, intervention details, observation characteristics, outcome measures, and key findings. Intervention details included simulation modality or topic, role allocation, and debriefing participation; observation characteristics included on-site versus off-site observation and directed versus non-directed observation. Outcome data were subsequently categorized according to the Kirkpatrick evaluation model [11].

### Study risk-of-bias assessment

Potential sources of bias were assessed qualitatively by 2 reviewers (A.A. and K.T.) by examining participant selection, role-allocation procedures, sample size justification, and sample representativeness. Disagreements were resolved by consensus. Methodological quality was assessed separately using the Medical Education Research Study Quality Instrument (MERSQI) [14], with interrater reliability assessed using the kappa statistic (κ) [15].

### Effect measures

Because meta-analysis was not conducted owing to methodological heterogeneity across the included studies, results are presented narratively. For each outcome, between-group differences are reported using the effect measures presented in the original studies, including mean differences, standardized mean differences, and effect sizes such as Cohen’s d and partial η², where available. Findings were considered statistically significant at P<0.05.

### Synthesis methods

Two authors (A.A. and K.T.) conducted data synthesis through the extraction, classification, and tabulation of quantitative findings to address the aim of this review. Each study was examined multiple times to ensure more precise data extraction. Any disagreements were resolved through consultation with a co-author (L.L.). A narrative synthesis was conducted to summarize the impact of learner role—active participant or observer—on learning outcomes during SBE.

### Reporting bias assessment

A formal assessment of reporting bias was not conducted because the review was designed as a narrative synthesis, and no quantitative synthesis was planned. Therefore, methods for assessing publication bias, such as funnel plot analysis, were not applicable. Potential reporting-related limitations, including the restriction to peer-reviewed English-language studies and the exclusion of grey literature, were considered when interpreting the findings.

### Certainty assessment

A formal certainty-of-evidence assessment was not performed because the heterogeneity of study designs, outcome constructs, and measurement instruments, combined with the narrative synthesis approach and absence of pooled effect estimates, precluded its valid application.

## Results

### Study selection

**Fig. 1.**
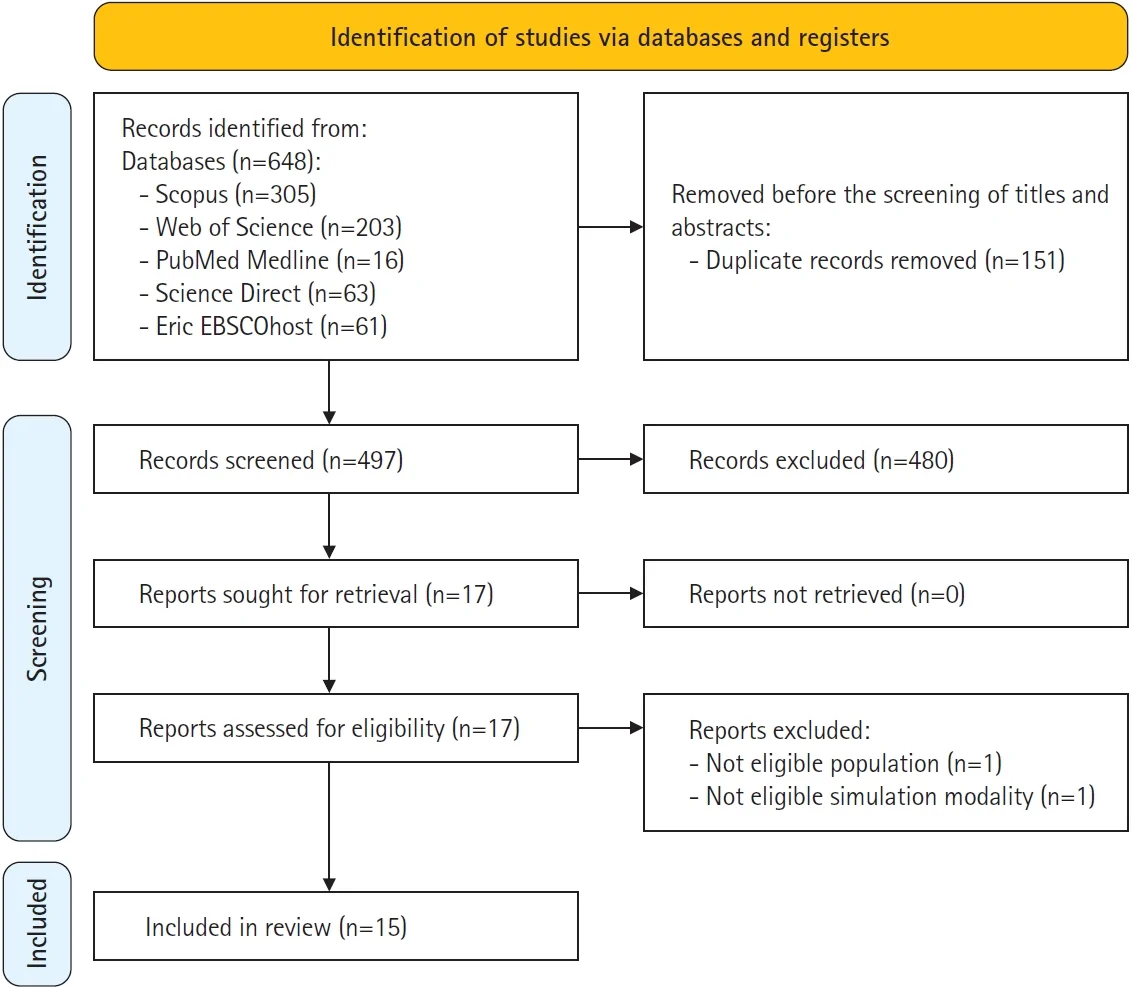
PRISMA (Preferred Reporting Items for Systematic Reviews and Meta-Analyses) flow diagram of the selected studies.

The search strategy identified 648 studies. Following deduplication using the Rayyan platform [13], 497 studies remained for title and abstract screening, of which 480 were excluded for ineligibility based on study purpose. Seventeen studies were retrieved for full-text review, of which 2 were excluded. Finally, 15 studies were included in this systematic review [1,10,16-28]. Fig. 1 presents the study selection process using a PRISMA flow diagram.

### Study characteristics

The 15 included studies were published between April 2018 and November 2024 [1,10,16-28]; one-third (5/15) were published in 2024 [1,19-22]. Most studies were conducted in high-income countries (13/15), with a predominance of studies from the United States (n=8) [10,16,18,21,23-25,28], and only 2 were conducted in an upper-middle-income country, Brazil [19,26].

Study designs included randomized controlled trials (n=3) [1,16,17], quasi-experimental designs (n=7) [10,18,19,21,24,27,28], mixed-methods designs (n=2) [20,22], an experimental design [23], and observational designs [25,26]. Most studies were single-center studies; only 2 were multicenter [18,23].

Twelve studies reported the use of at least one validated and reliable instrument [1,10,16-18,20-22,24-26,28]. Across all 15 studies, 1,799 participants were enrolled, with sample sizes ranging from 28 [27] to 267 [21]. Most participants were nursing students (87.1%) [1,10,16,18-24,26,28]. A total of 644 active participants [1,10,16,18-24,26] and 709 observers [1,10,16-24] were reported. All participants were trainees.

Regarding observer characteristics, classified according to O’Regan et al. [2], observation location was categorized as on-site or off-site, and observation structure was categorized as directed or non-directed. Observers were assigned to a separate room and observed the simulation via audiovisual feed in 8 studies [10,17-20,23-25], remained in the simulation room in 3 studies [16,21,22], and had an unspecified observation location in 4 studies [1,26-28]. Directed observation was used in 7 studies [1,10,16,22,24-26], non-directed observation was used in 5 studies [17,19,21,23,27], and observation structure was not specified in 3 studies [18,20,28]. All included studies were conducted in laboratory simulation settings. Simulation modalities included mannequin-based high-fidelity simulation (HFS; n=8) [1,10,16,17,19,21,23,28], HFS using standardized patients (SPs; n=3) [20,24,25], combined HFS with mannequins and SPs [22], SPs with low-fidelity mannequins [18,26], and combined HFS, SP, and telephone-based scenarios (n=1) [27]. All 15 studies included debriefing for both active participants and observers. Role allocation included fixed role assignment (n=9) [1,10,16,18,20-24], role-switching or rotation designs (n=4) [25-28], an active participant–observer hybrid design in which learners participated actively in one simulation and observed the remaining scenarios, compared with an observer-only group (n=1) [17], and one study in which participants were initially assigned to active participant or observer roles before all participants performed actively at follow-up without observers [19]. Regarding the theoretical frameworks underpinning the studies, only 3 studies did not report a conceptual framework [20,26,27]. Frameworks relevant to observational learning included Bandura’s social cognitive theory [1,18,19,23,25] and Johnson’s observational experiential learning framework [1,23]. Only 3 studies explicitly classified outcomes according to Kirkpatrick’s 4-level evaluation model [17,22,27]. The longest follow-up assessment period was 5 months [25].

### Methodological quality and potential sources of bias in studies

MERSQI scores for the included studies ranged from 9.5/18 [26] to 16/18 [23], with a mean±standard deviation of 12.70±1.55. Agreement was substantial (weighted κ=0.77) according to established thresholds [29]. Assessment results for all included studies are reported in Table 3.

Potential sources of bias were considered narratively. Participant selection was nonrandomized or not reported in all included studies, as most used convenience, voluntary, administrative, or curriculum-based recruitment [1,10,16-28]. In contrast, role allocation was reported as randomized in 10 studies [1,10,16-18,20,21,23,24,28], nonrandomized in 4 studies [19,22,25,26], and not specified in one study [27]. Although 11 of 15 studies performed a power analysis [1,10,17,18,20,21,23-27], 3 studies had small sample sizes [25-27]. Limited sample diversity may also have reduced representativeness and limited the generalizability of findings in 5 studies [16,18,20,21,24].

### Results of syntheses

Outcomes were categorized according to the 4 levels of Kirkpatrick’s evaluation model [11]. At Level 1, 8 studies [1,10,17,21,22,25,26,28] assessed reaction outcomes, including satisfaction [10,17,22,28], perceptions [21], anxiety [10], emotional arousal [25], and learning flow [1]. At Level 2, all 15 included studies assessed one or more learning outcomes across 3 subdimensions: knowledge, skills, and attitudinal outcomes. Knowledge outcomes encompassed acquisition [1,17,19,21-24,28], demonstration and application [23], and retention [17,19,23]. Skills outcomes included technical skills [19], clinical competency [28], clinical reasoning [1,16], medical communication and management [27], problem-solving and collaboration [10], and composite non-technical skills [17,19]. Attitudinal outcomes encompassed self-efficacy [18-20,22], self-confidence in learning [10,26,28], confidence in clinical practice [10], self-awareness [24], self-motivation, self-regulation, overall learning effectiveness [22], emotional intelligence, and coping with death [20]. No included study assessed Level 3 behavioral transfer to clinical practice or Level 4 patient outcomes. Perceived learning transfer, reported by one study [17], was reclassified as a Level 2 attitudinal outcome in the present review because it reflects self-reported beliefs about learning utility rather than directly observed behavioral change in real clinical practice, consistent with Kirkpatrick and Kirkpatrick’s [11] distinction between demonstrated behavior and self-reported learning.

#### Impact of learner role on Kirkpatrick Level 1 outcomes

Eight of the 15 studies examined Level 1 outcomes [1,10,17,21,22,25,26,28]. Among these, 5 studies specifically assessed satisfaction [10,17,22,26,28]. Most studies assessing satisfaction did not identify statistically significant between-role differences (P>0.05) [10,22,26]. However, Blanié et al. [17] reported higher satisfaction in the active participant–observer group than in the observer-only group (P=0.019), whereas Zulkosky et al. [28] reported high satisfaction scores across the 2 roles, with no statistically significant differences detected between conditions (deliberate practice vs. role switching) or scenarios (first vs. second) (P>0.79 and P>0.10, respectively). Perceptions of simulation were positive and did not differ significantly across roles (97% agreement; P=0.37) [21]. State anxiety decreased significantly in both roles after SBE (P<0.01), with no statistically significant between-role differences at pretest (P=0.432) or posttest (P=0.204) [10]. Learning flow improved in both roles, with no statistically significant difference between active participants and observers (P=0.291) [1]. Finally, emotional arousal was the only Level 1 outcome favoring active participants over observers, with higher negative and positive arousal in both uniprofessional and multiprofessional contexts (all P<0.001) [25].

#### Impact of learner role on Kirkpatrick Level 2 outcomes

Knowledge acquisition was measured immediately after simulation in 8 studies [1,17,19,21,22,24,25,28]. All 8 studies demonstrated significant within-role improvement; 7 studies did not identify statistically significant between-role differences. The exception was one study [17], in which the active participant–observer group outperformed the observer-only group (P=0.001). Immediate knowledge application, evaluated only by Johnson [23] using a validated, author-developed instrument operationalizing assimilation and accommodation, did not differ significantly between roles (P=0.459). Knowledge retention was evaluated at delayed follow-up in 4 studies [17,19,23,25], one of which was uninterpretable owing to insufficient statistical power [25]. Across the remaining 3 studies [17,19,23], knowledge decay was not uniform across roles or time points: Johnson [23] reported a significant 4-week decline in both roles with no between-role difference (P=0.593); de Moraes et al. [19] identified a selective significant 14-day decline among active participants (P<0.05); and Blanié et al. [17] found a significant 3-month decline only in the active participant–observer group (P=0.001), with no between-group difference (P=0.277).

Regarding the skills subdimension of Level 2, 6 studies assessed technical and clinical performance outcomes [1,10,16,19,27,28], 2 evaluated composite non-technical skills [17,19], and one assessed non-technical skills-related affective outcomes [20]. Across these skills-related outcomes, clinical reasoning (P=0.576 [1]; no inferential statistic reported [16]), clinical ability (P=0.721) [10], medical management (P>0.1), and overall performance (P=0.36) showed no statistically significant between-role differences [27]. Likewise, medical communication did not improve with prior simulation exposure in either role [27]. Composite non-technical skills findings showed no consistent active participant advantage over observers across studies (P>0.05 [17]; non-technical skills performance was described as not significantly different in the original study despite a reported P=0.044 [19]). In contrast, Esteban-Burgos et al. [20] reported improvement in coping with death and emotional intelligence in both roles (P<0.01), with greater improvement among active participants in coping with death (P=0.006), emotional attention (P=0.009), and emotional repair (P=0.006). Furthermore, 2 findings favored active participants: higher technical skills at 14-day follow-up (P=0.047) [19] and better performance among learners maintaining the active participant role across repeated scenarios (P=0.003) [28]. Overall, skills-related outcomes most often showed no statistically significant between-role differences, with active participant advantages limited to non-technical skills-related affective outcomes [20], delayed technical skills [19], and role-continuity effects on clinical competency [28].

Eight studies assessed attitudinal outcomes [10,18-20,22,24,26,28]. Self-efficacy improved significantly in both roles across 4 studies [18-20,22], with no statistically significant between-role differences in 3 studies (P>0.05 [18]; P=0.102 [20]; P=0.283 [22]); however, one study reported higher self-efficacy among active participants than observers (P<0.05) [19]. Self-confidence in learning did not differ significantly between roles across 2 studies (P=0.374 [10]; P>0.05 [26]), nor did confidence in clinical practice (P=0.710) [10]. Zulkosky et al. [28] similarly reported no significant between-role differences in self-confidence across role-assignment conditions (P>0.57) or scenarios (P>0.29), with no condition-by-scenario interaction (P>0.75). Self-regulation, self-motivation, and overall learning effectiveness exhibited no statistically significant differences across roles (P=0.648, P=0.633, and P=0.847, respectively) [22]. Emotional intelligence and ability to cope with death improved in both roles (P<0.01), with selective active participant advantages in ability to cope with death (P=0.006), emotional attention (P=0.009), and emotional repair (P=0.006); emotional clarity showed no significant difference between roles (P>0.05) [20]. Perceived learning transfer did not differ significantly immediately after simulation (P=0.701) but was significantly higher in the active participant–observer group than in the observer-only group at 3 months (P=0.036) [17].

## Discussion

### Interpretation

This systematic review evaluated the impact of learner role—active participant versus observer—on learning outcomes in healthcare SBE using the Kirkpatrick evaluation model. Across Levels 1 and 2, most included studies did not identify statistically significant differences between roles in reactions, knowledge, clinical performance, non-technical skills, or most attitudinal outcomes. No evidence was identified at Kirkpatrick Levels 3 or 4, representing a persistent gap in role-comparison simulation research. Consequently, this review provides evidence comparing active participants and observers in terms of educational outcomes at Kirkpatrick Levels 1 and 2 rather than behavioral transfer to clinical practice or patient- and organization-level outcomes. These findings should be interpreted in light of the heterogeneous role-allocation structures and observation characteristics across the included studies.

The heterogeneity of role-allocation strategies conditions the extent to which a true role-specific effect can be detected and may partly account for the variability observed across studies. In fixed-role designs, learners remain either active participants or observers throughout the simulation, allowing learning outcomes to be attributed exclusively to each role and permitting a more precise comparison between the effect of hands-on participation and that of reflective observation. In contrast, role-switching and rotation designs may introduce sequence effects and carryover bias, as prior exposure as either an observer or an active participant may influence subsequent performance; such cumulative exposure, regardless of the specific role assigned, was found to enhance medical management and global performance relative to unexposed novices [27]. Moreover, the active participant–observer hybrid design may further modify role-specific effects, as the active group combined active participation in one scenario with observation in the others rather than representing pure active participation, compared with an observer-only group [17]. Accordingly, the role effects reported in this review should be interpreted in light of the specific role-allocation strategy used to avoid attributing design-related effects to the learner role itself.

The instructional design of the observer experience may partly account for the present findings. Directed observation supported by checklists or role-specific forms may enhance active watching and listening by orienting observers toward defined learning objectives [2]. Indeed, observer learning and satisfaction appear closely associated with observation tools and role clarity; notably, studies that supported observers with a structured tool reported outcomes as good as, or better than, those of active participants [2]. O’Regan et al. [2] interpreted this finding through Bandura’s social learning theory, whereby learning may occur vicariously through observation of others’ behaviors and their consequences [30]. Moreover, all included studies involved observers in debriefing, the key simulation phase in which essential learning occurs through reflection [31]. Debriefing may further optimize observer learning [2]: it operationalizes the reflective observation phase of the experiential learning cycle, transforming observed experience into learning [7], while facilitated feedback supports the correction of performance gaps [32]. This shared participation in debriefing may contribute to the absence of statistically significant between-role differences in learning outcomes. The observation setting may likewise shape engagement: on-site observation may afford direct sensory access and sustain engagement [21], whereas the physical co-presence of observers may increase discomfort among active participants and potentially affect their performance. Taken together, the apparent absence of statistically significant between-role differences may partly reflect variation in observer design rather than role assignment alone.

### Comparison with previous studies

#### Kirkpatrick Level 1

Most included studies did not identify statistically significant differences between active participants and observers at Kirkpatrick Level 1. This pattern is consistent with Delisle et al. [4], whose meta-analysis reported no significant pooled between-role difference in reaction outcomes among learners undergoing both technical and non-technical skills training. The present review extends this evidence by identifying no statistically significant between-role differences across additional reaction outcomes, specifically perceptions of learning and learning flow. These findings suggest that affective responses generally did not differ statistically between roles. The reduction in anxiety after SBE in both roles [10] may be explained by guided observation, as prior evidence indicates that defined observer roles can reduce stress and support connections between theory and practice [33]. Two exceptions favored active participants: satisfaction and emotional arousal. The higher satisfaction reported in one study [17] should be interpreted in the context of its active participant–observer hybrid design, which was unique to that study. This finding aligns with previous evidence indicating that learners may perceive the observer role as less engaging or monotonous and may therefore prefer active participation [34], although such preference does not reliably predict learning effectiveness. Emotional arousal was higher among active participants than observers across simulation contexts [25]. This finding is supported by physiological evidence showing increased salivary cortisol levels only among learners in the active participant role [35] and by previous research reporting greater emotional arousal and anxiety among hands-on learners than observers across different simulation designs [36]. This increased arousal likely reflects the greater cognitive load and experiential immersion associated with active participation, particularly among novice learners [4].

#### Kirkpatrick Level 2

Across knowledge, skills, and attitudinal domains, most included studies did not identify statistically significant differences between observers and active participants. This finding extends the conclusions of O’Regan et al. [2] but partly diverges from Delisle et al. [4], who reported a significant active participant advantage in skills outcomes. It is also consistent with a scoping review of 28 nursing simulation studies reporting measurable observer learning in knowledge, clinical judgment, and teamwork, predominantly within the cognitive domain [37]. The present review further acknowledges that the advantages associated with active participation were domain-specific and time-dependent, emerging primarily in delayed retention of technical skills rather than consistently across learning outcomes.

The absence of significant between-role differences in knowledge acquisition is consistent with prior evidence [2,4]. Moreover, the knowledge decay observed across both roles in most included studies may reflect a broader process of attrition affecting theoretical knowledge and practical performance alike [38]. The absence of statistically significant between-role differences in most non-technical skills outcomes is consistent with previous findings suggesting that directed observation, when combined with structured debriefing, may support non-technical skills development that does not differ significantly from that of active participants [2,35]. Consistent with this, a scoping review concluded that scaffolding observer activities, notably through clinical decision-making models, may optimize observer engagement and the development of clinical reasoning [39], and further evidence indicates that directed observation may support clinical judgment development in the observer role [40]. The delayed technical-skills advantage observed among active participants [19] contrasts with previous findings showing no statistically significant between-role difference in technical-skills performance at follow-up, suggesting that any initial active participant advantage may not persist over time and that long-term outcomes may not differ significantly between roles [4]. Attitudinal outcomes generally did not differ significantly between roles across studies, consistent with the possibility that structured engagement and inclusive debriefing may support affective and motivational learning in both roles [2].

### Limitations

This review has several limitations. First, conclusions are based mainly on the reported absence of statistically significant between-group differences. Because no formal equivalence or noninferiority testing was performed and no meta-analysis or pooled effect estimate was generated, this absence should be interpreted with caution and does not establish equivalence between roles. Second, none of the studies assessed Kirkpatrick Levels 3 or 4, limiting conclusions regarding behavioral transfer to clinical practice and patient or organizational outcomes. Third, the lack of consistency in outcome measures, combined with limited longitudinal follow-up, restricted the interpretation of learning retention. Fourth, role-assignment strategies and observer conditions differed across studies, including fixed-role, hybrid, role-switching, and multistation formats; directed versus non-directed observation; observer location; and use of structured observation tools. This heterogeneity limited interpretation and the precision of aggregated comparisons. Finally, the evidence was concentrated in single-center studies, high-income countries, and nursing programs, and the restriction to peer-reviewed English-language publications may have introduced publication and language bias, limiting the generalizability and comprehensiveness of the evidence base.

### Implications

#### Implications for policymakers and educators in health professions education

This review provides preliminary empirical support for recognizing the observer role as a purposeful pedagogical approach in SBE. Integrated alongside active participation, the observer role may allow learners to benefit from observational learning, offering a cost-effective and resource-efficient response to the logistical and staffing constraints involved in engaging large cohorts in hands-on simulation. Nevertheless, realizing this potential requires investment in faculty development and audiovisual infrastructure to support high-quality observer engagement. Accordingly, policymakers, in collaboration with professional simulation societies, are encouraged to develop best-practice standards for learner role assignment, particularly for the observer role.

For educators, these results reframe the observer role from a logistical compromise to an intentional teaching strategy, as observers may achieve outcomes that do not differ significantly from those of active participants across several domains, although formal equivalence has not been established. Observer roles should therefore be designed with defined learning objectives, validated observation tools, and active involvement in debriefing. Furthermore, the decline observed in knowledge and technical skills at follow-up points to the importance of refresher sessions and spaced repetition in both roles to sustain learning.

#### Implications for future studies

These findings underscore several priorities for future research. First, longitudinal, multicenter studies are needed to compare learning outcomes between roles at Kirkpatrick Levels 3 and 4. Second, future studies should use objective, faculty-rated instruments for skills assessment and further examine reaction outcomes such as anxiety, learning flow, and perceived task load. Third, learner perceptions of the observer role should be explored as potential moderators of learning outcomes. In addition, the effect of observers remaining on-site alongside active participants versus off-site warrants further investigation, as it may influence both observer engagement and the performance of active participants. Future role-comparison studies should also involve more diverse health professions, including medicine, midwifery, social work, pharmacy, allied health, postgraduate trainees, and continuing professional development practitioners. Moreover, further research in in situ simulation is needed to determine whether learner-role effects vary across simulation contexts. Finally, studies should explicitly report role-allocation structure, observation modality, guided observation, theoretical framework, and targeted Kirkpatrick level to support more rigorous evidence synthesis.

## Conclusion

The findings of this review indicate that, at Kirkpatrick Levels 1 and 2, most included studies did not identify statistically significant differences in educational outcomes between observers and active participants. However, because the evidence was derived predominantly from undergraduate nursing students in high-income settings, these results should be interpreted with caution and should not be generalized to all health professions, training levels, or low- and middle-income settings. Further comparative studies of learner roles are needed to evaluate Kirkpatrick Levels 3 and 4, particularly behavioral transfer to clinical practice and patient- or organization-level outcomes. Within this scope, the observer role, particularly when structured or directed, may represent a scalable pedagogical strategy for expanding access to SBE when active participation opportunities are limited.

## Figures and Tables

**Figure f2-jeehp-23-24:**
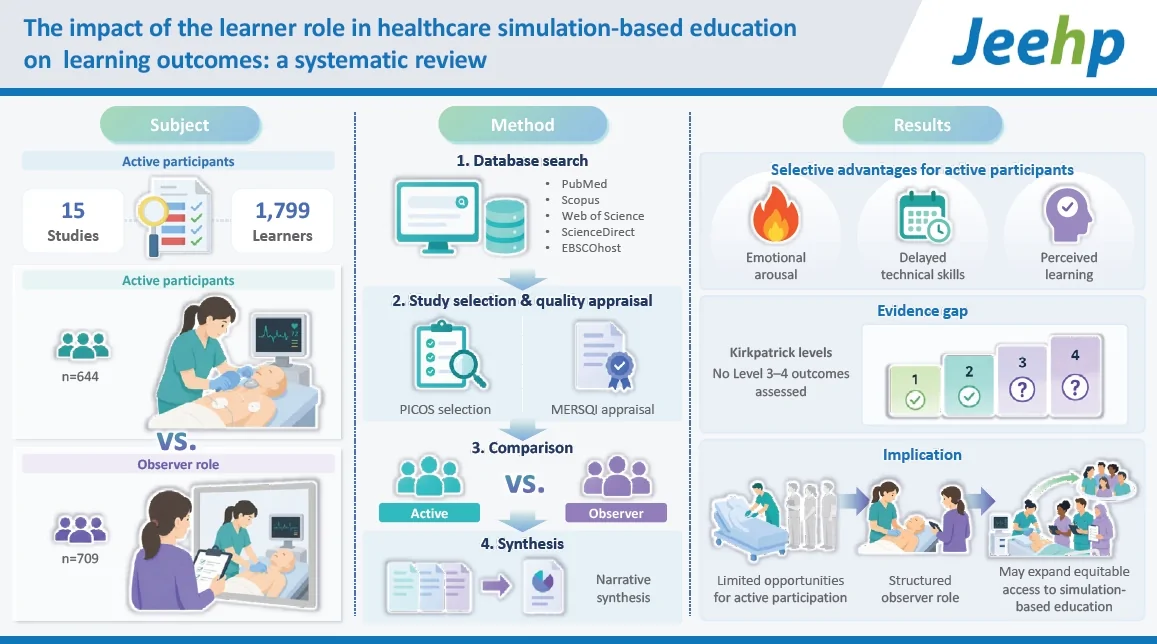


**Table 1.** Eligibility criteria

Criterion	Description of inclusion criteria
Population	Health professions students and practitioners at any level of training enrolled in formal simulation-based education programs, including nursing, medicine, and allied health disciplines.
Intervention	Healthcare simulation-based education with debriefing using mannequin-based simulation at any fidelity level, standardized patient scenarios, or role-play simulation.
Comparison	Active participant versus observer roles during simulation, including all role-allocation strategies and observation characteristics.
Outcomes	Comparative learning outcomes, including reactions, learning, behaviors, and patient outcomes, between active participants and observers during the simulation experience.
Study design	Randomized controlled trials, quasi-experimental designs, case-control studies, cross-sectional studies, prospective cohort studies, observational studies, and mixed-methods studies with extractable quantitative data.

**Table 2.** Summary of selected studies

Author (year), country	Study design	Study’s framework	Participants (N); trainee vs. professional; APs (n); observers (n)	Intervention (simulation modality/topic; role allocation; debriefing participation)	Observation characteristics		Quantitative outcomes (Kirkpatrick 4 levels)	Key findings
					On-site vs. off-site	Directed vs. non-directed		
Blanié et al. [17] (2018), France	Prospective RCT	Kolb’s experiential learning cycle (1984); Ericsson’s DP theory (2004); Kirkpatrick’s evaluation model	Anesthesia residents (N=104); trainees; AP-observer (AP-O) (n=59); observers (n=45)	HFS: 4 anesthesia CRM scenarios; random allocation to AP-observer (AP-O) or observer only; shared debriefing	Off-site	NDO	L1: satisfaction with learning	Immediate: Satisfaction higher in AP-O group (P=0.019). Knowledge higher in AP-O role (P=0.001). No significant between-role difference in NTS (P>0.05) and in learning transfer (P=0.701).
							L2: knowledge acquisition/retention, non-technical skills, perceived learning transfer	3 months: Knowledge decay occurred only in AP-O group (P=0.001) with no between-group difference (P=0.277). NTS declined in both roles (P<0.05) with no between-role difference (P>0.05); learning transfer was higher among AP-O than observers (P=0.036).
Hooper & Carlson [21] (2024), USA	Quasi-experimental, 1-group pre-post	NLN Jeffries simulation theory (2021)	Nursing students (N=267); trainees; APs (n=135); observers (n=132)	HFS: blood products administration scenario; random AP/observer allocation; shared debriefing	On-site	NR	L1: student perception	Immediate: Knowledge improved in both roles (P<0.001), with no statistically significant between-role difference (P=0.84). Perceptions were positive in both roles (97% agreed/strongly agreed), with no statistically significant role difference (P=0.37).
							L2: knowledge acquisition	
de Moraes et al. [19] (2024), Brazil	Quasi-experimental pre/posttest	Bandura’s social learning & self-efficacy theories	Medical and nursing undergraduates + residents (N=44); trainees; APs (n=22); observers (n=22)	HFS: palliative extubation; self-selected roles; shared debriefing	Off-site	NDO	L2: technical/NTS, knowledge acquisition/retention, and self-efficacy	Immediate: Knowledge improved in both roles, with no between-group difference (P>0.05). APs scored higher in self-efficacy (14.77 vs. 14.00, respectively), with statistically significant between-role difference (P<0.05).
								14 days: Knowledge decay occurred only in APs (P<0.05), with no between-group difference (P>0.05). APs outperformed observers in technical skills (P=0.047). NTS did not favor APs over observers (P=0.044). APs had higher mean self-efficacy than observers (14.54 vs. 13.51).
Esteban-Burgos et al. [20] (2024), Spain	Non-blind RCT and qualitative study	NR	Nursing students (N=264); trainees; observers (n=113); APs (n=51); control group (n=100)	SP simulation using trained actors: palliative care scenarios; 2 students randomly selected as APs per group; remaining participants assigned to observers; shared debriefing	Off-site	NR	L2: coping with death, emotional intelligence, and self-efficacy	Immediate: All outcomes improved in both roles (P<0.01). APs showed greater improvement in coping with death (P=0.006), emotional attention (P=0.009), and emotional repair (P=0.006). No significant between-group difference in self-efficacy (P=0.102) or emotional clarity (P>0.05).
Jiang et al. [22] (2024), Taiwan	Prospective mixed-methods	Kolb’s experiential learning theory; Kirkpatrick’s 4-level evaluation model	Nursing students (N=134); trainees; APs (n=67); observers (n=67)	HFS: 3 emergency scenarios; role self-selection (≤50% performers); shared facilitator debriefing with observer feedback	On-site	Directed	L1: satisfaction	Immediate: Knowledge improved in both roles (P<0.001), with no statistically significant role difference (P=0.550). No statistically significant between-role differences were reported for satisfaction (P=0.513), self-motivation (P=0.633), self-regulation (P=0.648), self-efficacy (P=0.283), and overall learning effectiveness (P=0.847).
							L2: knowledge, learning effectiveness (self-motivation, self-regulation, self-efficacy)	
Johnson [23] (2019), USA	Experimental, pretest-multiple posttests, repeated-measures	Kolb’s experiential learning theory (2015); Bandura social learning (1971); Johnson observational experiential learning (2018)	Nursing students (N=119); trainees; APs (n=59); observers (n=60)	HFS: opioid-induced respiratory depression + parallel anaphylaxis case; random role assignment; shared debriefing.	Off-site	NDO	L2: knowledge demonstration, retention and application	Immediate: Knowledge demonstration improved significantly in both roles post-simulation and post-debriefing (P<0.0005), with no statistical between-role difference at any timepoint (post-simulation: P=0.406; post-debriefing: P=0.673; overall: P=0.773).
								No statistically significant between-role difference in knowledge application at post-debriefing (P=0.459).
								At 4 weeks: Knowledge retention declined significantly in both roles (P<0.0005) with no statistically significant between-role difference (P=0.593); No significant between-role difference in knowledge application (P=0.446)
Ying et al. [27] (2020), Canada	Quasi-experimental	NR	Surgical residents (N=28); trainees; AP; observer (NR)	Mixed modality simulation (14 stations); APs active in 2–4 stations; observers watched remaining; shared debriefing	On-site	NDO	L2: medical management, communication, overall performance	Immediate: Medical management and overall performance were significantly higher in both APs and observers than in the no-exposure group (3.24 and 3.06 vs. 2.72; P˂0.02); with no between-role difference for either outcome (P>0.1 and P=0.36, respectively). Communication did not improve in either role.
Bates et al. [10] (2019), USA	Quasi-experimental	NLN Jeffries simulation theory	Nursing students (N=132); trainees; APs (n=66); observers (n=66)	HFS: medication administration and symptom management; random role allocation; shared debriefing	Off-site	Directed	L1: satisfaction, state anxiety	Immediate: State anxiety decreased in both roles (APs: P<0.001; observers: P<0.001), with no statistically significant between-role difference at pre-simulation (P=0.432) and post-simulation (P=0.204). Satisfaction, clinical ability, problem-solving, collaboration, self-confidence, and confidence in clinical practice did not differ significantly between roles (all P>0.05).
							L2: clinical ability, problem-solving, collaboration, self-confidence, confidence in clinical practice	
Alexander [16] (2020), USA	Double-blinded, RCT	Clinical reasoning model; Felder-Silverman learning style model	Nursing students (N=204); trainees; APs (n=117); observers (n=87)	Nursing clinical scenarios: acute care management; role assignment by learning style congruence; shared debriefing	On-site	Directed	L2: clinical reasoning	Immediate: Clinical reasoning improved significantly in both roles (APs: 55.68 → 59.42, P<0.001; Observers: 55.21 → 59.10, P<0.001). The 2 roles achieved similar learning outcomes.
Zulkosky et al. [28] (2021), USA	Mixed factorial + quasi-experimental	NLN Jeffries simulation theory; DP framing	Nursing students (N=159); trainees; AP; observers (NR)	HFS: pediatric critical care; randomized to DP vs. role switching and to AP vs. observer within groups; shared debriefing	NR	NR	Level 1: satisfaction	Immediate: No statistically significant differences in satisfaction were reported across conditions and scenarios (P>0.79; P>0.10, respectively). Knowledge improved (P<0.001); no condition or role effect (P>0.67; P>0.56). Clinical competency improved from scenario 1→ 2 (P<0.001); DP outperformed role switching at run 2 (P=0.003). No statistically significant differences in self-confidence were reported across role switching or DP (P>0.57).
							Level 2: knowledge acquisition, clinical competency, self-confidence in learning	
Teixeira et al. [26] (2022), Brazil	Observational within-subject study	NR	Nursing students (N=44); AP; observer (NR, role switching design)	Realistic simulation with SP + low-fidelity mannequin; within-subject role switching (actor in 2 scenarios; observer in 2); shared debriefing	NR	Directed	L1: satisfaction in learning	Immediate: satisfaction and self-confidence did not differ significantly between APs and observers (P>0.05)
							L2: self-confidence in learning	
Rogers et al. [25] (2019), USA	Prospective cohort study	Kolb’s experiential learning theory (1984); Bandura social learning theory (1962); Circumplex model of emotions	Pediatric residents (N=56); trainees; AP; observer (NR, rotation design)	Two simulation programs: pediatric emergency + multidisciplinary cardiac arrest; mixed role rotation; shared debriefing	Off-site	Directed	L1: emotional arousal	Immediate: APs reported significantly higher negative arousal than observers in uniprofessional (F(1,24)=33.8, P<0.001) and multiprofessional simulations (t(32)=4.96, P<0.001). Positive arousal was also higher among APs in both contexts (F(1,24)=16.2, P=0.001; t(32)=4.86, P<0.001). No statistically significant between-role difference in knowledge was reported.
							L2: knowledge acquisition and retention	5-month follow-up: No detectable role effect on knowledge retention (multivariable clustered regression; coefficient=0.07; 95% CI, −0.21 to 0.35).
Kirkpatrick et al. [24] (2020), USA	Quasi-experimental 1-group repeated measure (pretest/posttest) study	Shared theory in palliative care; compassionate holistic attentive, adaptable realistic/resolute and moral concept model	Nursing students (N=75); trainees; APs (n=36); observers (n=39)	End-of-life simulation using SP (dying patient + family member); role assignment by card draw; shared debriefing	Off-site	Directed	L2: knowledge acquisition, self-awareness	Immediate: Knowledge improved in both roles (APs: 56.2% to 80.5%; observers: 57.6% to 77.9%; P<0.001), with no statistically significant between-role difference (P=0.248). Self-awareness increased in both roles (P<0.001), with no statistically significant between-role difference at posttest (P=0.316).
Ham et al. [1] (2024), Republic of Korea	Parallel RCT (2-group pretest-posttest)	Bandura’s social learning theory (1977); Kolb’s experiential learning theory (1984); Johnson’s observational experiential learning framework (2020)	Nursing students (N=69); trainees; APs (n=37); observers (n=32)	HFS: hip arthroplasty nursing care; random allocation by number draw; shared debriefing	NR	Directed	L1: learning flow	Immediate: Learning flow, knowledge, and clinical reasoning improved significantly in both roles, with no statistically significant between-group differences for learning flow (P=0.291), knowledge (P=0.917), or clinical reasoning (P=0.576).
							L2: knowledge acquisition; clinical reasoning	
Clark & Lippe [18] (2022), USA	Quasi-experimental (2 group repeated measures)	Bandura’s social cognitive theory + self-efficacy theory (1977, 1986, 1994)	Nursing students (N=100); trainees; APs (n=54); observers (n=46)	Pediatric end-of-life communication simulation using low-fidelity manikin + SP; random role assignment; shared debriefing	Off-site	NR	L2: communication self-efficacy	Immediate: Communication self-efficacy improved significantly over time in both roles (all time effects P<0.001); no statistically significant between-role difference was reported on 7/9 dimensions (P>0.05).

APs, active participants; HFS, high-fidelity simulation; SP, standardized patient; NDO, non-directed observation; NR, not reported; RCT, randomized controlled trial; NLN, National League for Nursing; L1, Kirkpatrick Level 1; L2, Kirkpatrick Level 2; CRM, crisis resource management; NTS, non-technical skills; DP, deliberate practice.

**Table 3.** Quality assessment of the included studies using the MERSQI tool

Study	Study design	Sampling	Type of data	Validity of evaluation instrument	Data analysis	Outcome	Total score/18
Blanié et al. [17] (2018)	3	2	3	1	3	1.5	13.5
Hooper & Carlson [21] (2024)	1.5	2	3	2	3	1.5	13
de Moraes et al. [19] (2024)	2	2	3	1	2	1.5	11.5
Esteban-Burgos et al. [20] (2024)	3	2	1	2	3	1	12
Jiang et al. [22] (2024)	2	2	3	2	3	1.5	13.5
Johnson [23] (2019)	3	2.5	3	3	3	1.5	16
Ying et al. [27] (2020)	1	2	3	1	3	1.5	11.5
Bates et al. [10] (2019)	2	2	1	3	3	1	12
Alexander [16] (2020)	3	1	1	2	3	1.5	11.5
Zulkosky et al. [28] (2021)	2	2	3	2	3	1.5	13.5
Teixeira et al. [26] (2022)	2	1.5	1	1	3	1	9.5
Rogers et al. [25] (2019)	1	1.5	3	3	3	1.5	13.5
Kirkpatrick et al. [24] (2020)	1.5	2	3	2	3	1.5	13
Ham et al. [1] (2024)	3	2	3	2	3	1.5	14.5
Clark & Lippe [18] (2022)	3	2.5	1	1	3	1	11.5

MERSQI, Medical Education Research Study Quality Instrument.

## Supplementary materials

Supplement 1. Search strategies for each database.

Supplement 2. Audio recording of the abstract.
