# Supplementary material for: The impact of the learner role in healthcare simulation-based education on learning outcomes: a systematic review
Source: J Educ Eval Health Prof. 2026 Aug 6;23:24. doi: 10.3352/jeehp.2026.23.24 (PMC13616528; doi:10.3352/jeehp.2026.23.24)
Supplement: Supplementary file 1 [file jeehp-23-24-suppl1.docx]

**Digital Supplement 1:** search strategies for database

| Data base | Search keywords and filters applied until november 29, 2024 |
| --- | --- |
| Scopus | TITLE-ABS ( ( "simulation" OR "healthcare simulation" OR "simulation-based learning" OR "simulation training" OR "simulation-based education" OR "simulation learning" ) ) AND ( TITLE-ABS ( "midwife" OR "nursing" OR "physician" OR "health student" OR "undergraduate student" OR "postgraduate student" OR "resident" OR "nursing student" OR "midwifery student" OR "healthcare learner" OR "healthcare trainee" OR "healthcare professional" ) ) AND ( TITLE-ABS ( "observer" OR "participant observation" OR "response-based role" OR "vicarious learning" OR "non-participant" OR "observer involvement" OR "learning by viewing" OR "hands-off" OR "active role" OR "active participant" OR "hands-on" ) ) AND ( TITLE-ABS ( "Kirkpatrick" OR "reaction" OR "satisfaction" OR "skills" OR "knowledge" OR "attitude" OR "behavior" OR "patient outcomes" ) ) AND PUBYEAR > 2017 AND PUBYEAR < 2026 AND ( LIMIT-TO ( SUBJAREA , "MEDI" ) OR LIMIT-TO ( SUBJAREA , "NURS" ) OR LIMIT-TO ( SUBJAREA , "HEAL" ) ) AND ( LIMIT-TO ( DOCTYPE , "ar" ) ) AND ( LIMIT-TO ( LANGUAGE , "English" ) ) |
| Web of science | (#1 AND #2 AND #3 AND #4) AND (DT==("ARTICLE") AND LA==("ENGLISH") AND SJ==("NURSING" OR "EDUCATION EDUCATIONAL RESEARCH" OR "SURGERY" OR "GENERAL INTERNAL MEDICINE" OR "NEUROSCIENCES NEUROLOGY" OR "HEALTH CARE SCIENCES SERVICES" OR "RADIOLOGY NUCLEAR MEDICINE MEDICAL IMAGING" OR "OBSTETRICS GYNECOLOGY" OR "CARDIOVASCULAR SYSTEM CARDIOLOGY" OR "ONCOLOGY" OR "ANESTHESIOLOGY" OR "ORTHOPEDICS" OR "EMERGENCY MEDICINE" OR "UROLOGY NEPHROLOGY" OR "PEDIATRICS" OR "DERMATOLOGY" OR "PHARMACOLOGY PHARMACY" OR "REHABILITATION" OR "RESEARCH EXPERIMENTAL MEDICINE" OR "OPHTHALMOLOGY" OR "OTORHINOLARYNGOLOGY" OR "RESPIRATORY SYSTEM")) |
| PubMed | ((("simulation"[Title/Abstract] OR "healthcare simulation"[Title/Abstract] OR "simulation-based learning"[Title/Abstract] OR "simulation training"[Title/Abstract] OR "simulation-based education"[Title/Abstract] OR "simulation learning"[Title/Abstract]) AND ("midwife"[Title/Abstract] OR "nursing"[Title/Abstract] OR "physician"[Title/Abstract] OR "health student"[Title/Abstract] OR "undergraduate student"[Title/Abstract] OR "postgraduate student"[Title/Abstract] OR "resident"[Title/Abstract] OR "nursing student"[Title/Abstract] OR "midwifery student"[Title/Abstract] OR "healthcare learner"[Title/Abstract] OR "healthcare trainee"[Title/Abstract] OR "healthcare professional"[Title/Abstract])) AND ("observer"[Title/Abstract] OR "participant observation"[Title/Abstract] OR "response-based |
| EricEbscot | (TI ("simulation" OR "healthcare simulation" OR "simulation-based learning" OR "simulation training" OR "simulation-based education" OR "simulation learning")) AND (TI ("observer" OR "participant observation" OR "response-based role" OR "vicarious learning" OR "non-participant" OR "observer involvement" OR "learning by viewing" OR "hands-off" OR "active role" OR "active participant" OR "hands-on")) OR (TI ("Kirkpatrick" OR "reaction" OR "satisfaction" OR "skills" OR "knowledge" OR "attitude" OR "behavior" OR "patient outcomes")) AND (TI ("midwife" OR "nursing" OR "physician" OR "health student" OR "undergraduate student" OR "postgraduate student" OR "resident" OR "nursing student" OR "midwifery student" OR "healthcare learner" OR "healthcare trainee" OR "healthcare professional")) |
| Science Direct | (("simulation") AND ("resident" OR "nursing student" OR "healthcare professional") AND ("observer” OR "active participant") AND ("Kirkpatrick")) |
